# The Grip Strength Loss Rate and the Subsequent Cognitive Decline Rate in Older Adults: The Moderating Role of Social Isolation

**DOI:** 10.1093/geroni/igae055

**Published:** 2024-06-04

**Authors:** Yanzhi Li, Liwan Zhu, Caiyun Zhang, Hao Zhao, Wanxin Wang, Lan Guo, Ciyong Lu

**Affiliations:** Department of Medical Statistics and Epidemiology, School of Public Health, Sun Yat-sen University, Guangzhou, People’s Republic of China; Guangdong Provincial Key Laboratory of Food, Nutrition and Health, Sun Yat-sen University, Guangzhou, People’s Republic of China; Department of Medical Statistics and Epidemiology, School of Public Health, Sun Yat-sen University, Guangzhou, People’s Republic of China; Guangdong Provincial Key Laboratory of Food, Nutrition and Health, Sun Yat-sen University, Guangzhou, People’s Republic of China; Department of Medical Statistics and Epidemiology, School of Public Health, Sun Yat-sen University, Guangzhou, People’s Republic of China; Guangdong Provincial Key Laboratory of Food, Nutrition and Health, Sun Yat-sen University, Guangzhou, People’s Republic of China; Department of Medical Statistics and Epidemiology, School of Public Health, Sun Yat-sen University, Guangzhou, People’s Republic of China; Guangdong Provincial Key Laboratory of Food, Nutrition and Health, Sun Yat-sen University, Guangzhou, People’s Republic of China; Department of Medical Statistics and Epidemiology, School of Public Health, Sun Yat-sen University, Guangzhou, People’s Republic of China; Guangdong Provincial Key Laboratory of Food, Nutrition and Health, Sun Yat-sen University, Guangzhou, People’s Republic of China; Department of Medical Statistics and Epidemiology, School of Public Health, Sun Yat-sen University, Guangzhou, People’s Republic of China; Guangdong Provincial Key Laboratory of Food, Nutrition and Health, Sun Yat-sen University, Guangzhou, People’s Republic of China; Department of Medical Statistics and Epidemiology, School of Public Health, Sun Yat-sen University, Guangzhou, People’s Republic of China; Guangdong Provincial Key Laboratory of Food, Nutrition and Health, Sun Yat-sen University, Guangzhou, People’s Republic of China

**Keywords:** Dementia, English Longitudinal Study of Ageing, Social isolation

## Abstract

**Background and Objectives:**

Accumulating evidence suggests that low grip strength (GS) is associated with a faster cognitive decline, but most previous studies have measured GS at a single time point, ignoring changes in GS. We aimed to explore the association of the GS loss rate with the sequent cognitive decline, as well as the moderating role of social isolation in older adults.

**Research Design and Methods:**

Data were from the English Longitudinal Study of Ageing. Absolute and relative GS loss rates were calculated as the annual losses from Wave 2 (2004–05) to Wave 4 (2008–09). Participants were divided into 3 groups according to the tertiles of GS loss rates. Linear mixed models were used to assess the association of the GS loss rate during Waves 2–4 with the cognitive decline rate during Waves 4–9 (Wave 9, 2018–19).

**Results:**

Of the 4 356 participants included in analyses, 1 938 (44.5%) were men, with a mean age of 68.4 (*SD*: 8.4) years. Compared with Tertile 1 of the absolute GS loss rate, Tertile 2 (β = −0.009 [95% CI: −0.018 to −0.001] *SD*/year) and Tertile 3 (β = −0.018 [95% CI: −0.027 to −0.010] *SD*/year) were associated with a faster cognitive decline rate. The results of relative GS were similar to those of absolute GS. Social isolation was a significant modifier in the associations of the absolute GS loss rate with decline rates in global cognition and episodic memory, but not in temporal orientation. We did not observe that social isolation moderated the association of the relative GS loss rate with the cognitive decline rate.

**Discussion and Implications:**

Both absolute and relative GS loss rates were positively associated with the cognitive decline rate in older adults. Low social isolation scores attenuated the association of the absolute GS loss rate with the cognitive decline rate.

**Translational Significance:** The routine monitoring of grip strength (GS) should be recommended among older adults, and those who have rapidly lost absolute or relative GS might be the high-risk population with rapid cognitive decline. Older adults should actively participate in physical exercise to reduce GS loss, and those who have lost GS quickly should participate in more social activities.

With the aging of the population, the number of individuals with dementia worldwide will rise from 57 million in 2019 to 153 million in 2050 (1). Dementia is characterized by a decline in cognitive function (2). Due to the lack of effective treatments, it is of great significance to identify modifiable risk factors to prevent or slow down cognitive decline (3). Accumulating evidence suggests that a lower grip strength (GS) at baseline is associated with a faster cognitive decline (4–8). GS can be measured easily and inexpensively, and most people can enhance it through physical exercise. Thus, GS shows outstanding potential for identifying the population at risk of rapid cognitive decline and formulating prevention strategies.

Grip strength (GS) usually declines with age among older adults (9), but most previous studies only measured GS at a single time point, ignoring GS loss with aging (4–8). A single GS measurement might be insufficient to predict subsequent cognitive decline. At present, several studies have explored the association of GS loss with cognitive decline in the same period (10–14), but it is unclear whether GS loss can predict subsequent cognitive decline because cognitive decline might also cause GS loss (15,16). Elucidating the association temporality can provide more nuanced insights for slowing down cognitive decline. To date, there is a lack of research exploring the correlation between the GS loss rate and the subsequent cognitive decline rate.

Hippocampal volume might play an important role in the association between GS and cognitive function (17,18). Social isolation is another factor related to cognitive function through hippocampal volume (19). Social isolation is a recognized modifiable risk factor for cognitive decline (20,21). Some studies have shown that a lower social isolation score can alleviate the association between some risk factors (eg, adverse childhood experiences) and cognitive decline (22). Nevertheless, it is unclear whether social isolation can moderate the correlation between the GS loss rate and the subsequent cognitive decline rate. Clarifying the moderating role of social isolation will bring enormous benefits to individuals who have lost GS quickly.

Using the data from the English Longitudinal Study of Ageing (ELSA), this prospective cohort study aimed to investigate the association between the GS loss rate and the subsequent cognitive decline rate in older adults. Additionally, the moderating role of social isolation in the above correlation was also explored.

## Method

### Study Design and Participants

We used the data from the ELSA, an ongoing, prospective, and nationally representative cohort of community-dwelling adults ≥50 years in the United Kingdom, which has been described previously in detail (23). Briefly, the ELSA was launched in 2002–03 (Wave 1), and participants were biennially followed up until 2018–19 (Wave 9). The ELSA received ethical approval from the London Multicenter Research Ethics Committee (MREC/01/2/91). All participants filled in the informed consent form.

Because GS was measured in Waves 2 and 4, the data from Waves 2 and 4 were used to calculate the annual loss rate of GS, and the data from Waves 4–9 were used to assess the cognitive decline rate. The timeline of this study is shown in Supplementary Figure 1. A total of 7 281 participants ≥50 years had the completed data on GS and weight in Wave 2. After excluding participants with missing data on GS or weight in Wave 4 (*n* = 2 508), with a memory-related disease in Wave 4 (*n* = 104), or lost follow-up during Waves 4–9 (*n* = 387), we eventually included 4 356 participants. Supplementary Figure 2 shows the inclusion and exclusion processes of participants.

### Assessment of Cognitive Function

In Waves 4–9, the ELSA assessed 3 dimensions of cognitive function, including episodic memory, executive function, and temporal orientation. The details of cognitive assessment have been previously reported (24–26). Episodic memory was evaluated by testing the immediate and delayed recall of 10 unrelated words. Episodic memory scores were the sum of words successfully recalled in the immediate and delayed word recall tests, ranging from 0 to 20. Executive function was assessed by a verbal fluency task in which participants were asked to list as many animal names as possible within 1 minute. The number of listed animal names was counted as executive function scores, without upper limits. Temporal orientation was evaluated by 4 questions on the current year, month, date, and week. The sum of correct answers was considered as temporal orientation scores, ranging from 0 to 4. For all tests, a higher score represents better cognitive performance. Moreover, the cognitive assessment date of each participant was recorded.

To evaluate the global cognitive function, standardized *z* scores were generated. First, we calculated *z* scores for each domain by subtracting the mean in Wave 4 and dividing it by the standard deviation (*SD*) in Wave 4. Then, we calculated the mean of the 3 *z* scores and used the same approach to obtain *z* scores for global cognitive function. A cognitive *z* score of −1 at any wave represents 1 *SD* below the mean cognitive scores in Wave 4. Such an approach has been widely used in previous studies (24–26).

### Assessment of the GS Loss Rate

In Waves 2 and 4, participants were asked to sit in an upright position with elbows on both sides and forearms bent at a 90° angle on the armrest. Three measurements of GS were taken on both the dominant and nondominant hand using a Smedley dynamometer. Participants who had a hand injury or surgery to either hand within the last 6 months did not participate in the GS test. The maximum recorded GS was used in the current analysis (27). Participants’ weight was measured using a portable electronic scale. Because the GS was closely related to weight, GS was expressed in absolute units (kg) and relative units (ie, GS divided by body weight, kg/kg) to represent the performance of the upper limb without and with correction of weight, respectively (28). In addition, the GS measurement date of each participant was recorded. The calculation formulas for the absolute and relative GS loss rates are as follows:


$$\begin{array}{lll} & Absolute\;GS\;loss\;rate\;=\; & \;\frac{\begin{array}{l} Absolute\;GS\;in\;Wave\;2\;\text{-}\;absolute\;GS\;in\;Wave\;4 \end{array}}{\begin{array}{l} GS\;measurement\;date\;in\;Wave\;4\;\text{-}\;GS\;measurement\;date\;in\;Wave\;2 \end{array}} \end{array}$$

$$\begin{array}{lll} & Relative\;GS\;loss\;rate\;=\; & \;\frac{\begin{array}{l} Relative\;GS\;in\;Wave\;2\;\text{-}\;relative\;GS\;in\;Wave\;4 \end{array}}{\begin{array}{l} GS\;measurement\;date\;in\;Wave\;4\;\text{-}\;GS\;measurement\;date\;in\;Wave\;2 \end{array}} \end{array}$$

To mitigate the impact of extreme values on correlations, explore nonlinear correlations, facilitate statistical description of baseline characteristics, and interpret results more clearly, we divided GS loss rates into 3 groups according to the tertiles: <0.001, 0.001–0.979, and >0.979 kg/year for the absolute GS loss rate; <0.001, 0.001–0.013, and >0.013 kg/(kg × year) for the relative GS loss rate, referring to previous literature and ensuring sufficient sample size for each group (29–32).

### Assessment of Social Isolation

In Wave 4, social isolation was assessed using the Social Isolation Index (33,34). This index included 5 dichotomized indicators: marital status (1 point for unmarried/not cohabiting), contacts with children (1 point for contacting in person or by telephone/written/e-mail less than monthly; 0 points for participants without children), contacts with other family members (1 point for contacting in person or by telephone/written/e-mail less than monthly), contacts with friends (1 point for contacting in person or by telephone/written/e-mail less than monthly), and social participation (1 point for not being a member of any organizations such as religious groups, gyms/sports clubs, and committees). Social isolation scores range from 0 to 5, and a higher score suggests greater social isolation.

### Assessment of Potential Covariates

All potential covariates were assessed in Wave 4, including age, sex (men or women), race/ethnicity (White race or others), educational level, employment status (retired or nonretired), smoking status (current smokers or noncurrent smokers), drinking status, physical activity, body mass index (normal and underweight [<25 kg/m^2^], overweight [25 to <30 kg/m^2^], or obese [≥30 kg/m^2^]), depressive symptoms (yes or no), self-reported diabetes (yes or no), self-reported hypertension (yes or no), and self-reported cardiovascular disease (yes or no). Educational level was categorized into high level (university degree or equivalent), middle level (A-level/higher education below degree), and low level (no qualifications/O-level or equivalent) (35). According to the frequency of drinking in the past 12 months, we categorized drinking status into less than weekly, 1−4 days a week, and 5−7 days a week (36). Physical activity was assessed by asking participants how often they participated in vigorous, moderate, and light physical activities, and was classified into vigorous, moderate, and light physical activities (37). Depressive symptoms were assessed using the 8-item version of the Center for Epidemiologic Studies Depression Scale. The total scores range from 0 to 8, with higher scores suggesting more severe depressive symptoms. A cut-off value of ≥4 was used to identify older adults with depressive symptoms (24).

### Statistical Analyses

Participants’ characteristics in Wave 4 were summarized according to the tertiles of the absolute GS loss rate. Data were shown as mean (*SD*) for continuous variables or frequency (percentage) for categorical variables and were compared using Pearson Chi-squared tests or 1-way analysis of variance, as appropriate.

In main analyses, we used linear mixed models with person-specific random intercepts to explore the association of the GS loss rate during Waves 2–4 with the cognitive decline rate during Waves 4–9 by including an interaction term between the GS loss rate and follow-up time (range: 0–11.08 years), with Tertile 1 as the reference. Crude models were first constructed, including the GS loss rate, follow-up time, the interaction term of the GS loss rate × follow-up time, and the random intercepts. The adjusted models additionally included age, sex, race/ethnicity, educational level, employment status, smoking status, drinking status, physical activity, body mass index, depressive symptoms, diabetes, hypertension, cardiovascular disease, GS, and social isolation in Wave 4. Results were presented as β coefficient and 95% confidence interval (CI). In addition, considering that converting continuous variables into categorical variables would result in loss of information, the GS loss rate was also treated as a continuous variable to repeat the above analyses.

When a significant association was observed, the secondary analyses were further conducted to explore whether social isolation moderates the associations of the GS loss rate with the cognitive decline rate by adding a 3-way interaction term of the GS loss rate × follow-up time × social isolation scores, with adjustment for the aforementioned covariates. To facilitate model interpretation for the 3-way interaction test and to ensure enough sample, the GS loss rate was used as a continuous variable in the above models.

We conducted a stratified analysis by age (50–64 years and 65–90 years) to explore the aforementioned associations across different age groups. In addition, we performed 3 sensitivity analyses to verify the robustness of the results. First, participants without children obtained 0 point for “contacts with children” in the assessment of social isolation in the main analysis. We excluded participants without children. Second, some covariates might vary with time, so we adjusted for time-varying covariates (ie, educational level, employment status, smoking status, drinking status, physical activity, body mass index, depressive symptoms, self-reported diabetes, self-reported hypertension, self-reported cardiovascular disease, and social isolation scores). Finally, relative GS (kg/[kg/m^2^]) was calculated by the ratio of GS strength to body mass index.

All statistical analyses were conducted using Stata version 17.0 (StataCorp LLC). Statistical significance was defined as a 2-tailed *p* < .05.

## Results

### Characteristics of Participants

We included 4 356 participants with a mean age of 68.4 (*SD*: 8.4) years in Wave 4, among whom 44.5% were men and 98.4% were White (Table 1). Compared with participants belonging to Tertile 1 of the absolute GS loss rate, participants belonging to Tertile 3 were older, were more likely to be men, retirees, frequent drinkers, and obese, did more vigorous physical activity, had a higher prevalence of diabetes and cardiovascular disease, and showed lower global cognition *z* scores, episodic memory *z* scores, temporal orientation *z* scores, absolute GS, and relative GS (all *p* < .05). The cumulative attrition rates of Waves 5, 6, 7, 8, and 9 were 1.8%, 7.9%, 19.5%, 30.0%, and 37.9%, respectively (Supplementary Figure 1).

**Table 1.** Characteristics of Participants in Wave 4 by the Absolute Grip Strength Loss Rate

Characteristic^a^	Total	The absolute grip strength loss rate			*p* Value^b^
		Tertile 1 (< 0.001 kg/y)	Tertile 2 (0.001–0.979 kg/y)	Tertile 3 (>0.979 kg/y)	
Participants, *n*	4 356	1 342	1 528	1 486	
Age, y, mean (*SD*)	68.4 (8.4)	67.1 (7.9)	68.7 (8.3)	69.2 (8.7)	<.001
Men	1 938 (44.5)	588 (43.8)	570 (37.3)	780 (52.5)	<.001
Race—White	4 288 (98.4)	1 321 (98.4)	1 502 (98.3)	1 465 (98.6)	.816
Educational level					.966
High	1 465 (33.6)	458 (34.1)	517 (33.8)	490 (33.0)	
Middle	355 (8.2)	111 (8.3)	124 (8.1)	120 (8.1)	
Low	2 536 (58.2)	773 (57.6)	887 (58.1)	876 (58.9)	
Retired	2 836 (65.1)	817 (60.9)	1 025 (67.1)	994 (66.9)	<.001
Current smokers	505 (11.6)	172 (12.8)	163 (10.7)	170 (11.4)	.195
Drinking status					.018
Less than weekly	1 490 (34.2)	506 (34.1)	533 (34.9)	451 (33.6)	
1−4 days a week	1 537 (35.3)	483 (32.5)	553 (36.2)	501 (37.3)	
5−7 days a week	1 329 (30.5)	497 (33.4)	442 (28.9)	390 (29.1)	
Physical activity					.020
Light	888 (20.4)	291 (21.7)	310 (20.3)	287 (19.3)	
Moderate	1 981 (45.5)	621 (46.3)	717 (46.9)	643 (43.3)	
Vigorous	1 487 (34.1)	430 (32.0)	501 (32.8)	556 (37.4)	
Body mass index					.416
Normal and underweight (<25 kg/m^2^)	1 100 (25.3)	336 (25.0)	409 (26.8)	355 (23.9)	
Overweight (25 to <30 kg/m^2^)	1 840 (42.2)	578 (43.1)	625 (41.0)	637 (42.9)	
Obese (≥30.0 kg/m^2^)	1 416 (32.5)	428 (31.9)	494 (32.3)	494 (33.2)	
Depressive symptoms	532 (12.2)	154 (11.5)	200 (13.1)	178 (12.0)	.396
Hypertension	1 840 (42.2)	533 (39.7)	652 (42.7)	655 (44.1)	.059
Diabetes	461 (10.6)	138 (10.3)	135 (8.8)	188 (12.7)	.003
Cardiovascular disease	1 030 (23.7)	282 (21.0)	353 (23.1)	395 (26.6)	.002
Global cognition *z* scores, mean (*SD*)	0.00 (1.00)	0.06 (0.98)	0.01 (1.01)	−0.06 (1.00)	.005
Episodic memory *z* scores, mean (*SD*)	0.00 (1.00)	0.09 (1.00)	−0.01 (1.01)	−0.07 (0.99)	<.001
Executive function *z* scores, mean (*SD*)	0.00 (1.00)	0.00 (1.00)	0.01 (1.00)	−0.01 (1.00)	.912
Temporal orientation *z* scores, mean (*SD*)	0.00 (1.00)	0.04 (1.00)	0.02 (1.00)	−0.06 (1.00)	.019
Absolute grip strength, kg, mean (*SD*)	30.85 (11.21)	34.23 (11.44)	29.84 (10.31)	28.85 (11.21)	<.001
Relative grip strength, kg/kg, mean (*SD*)	0.40 (0.13)	0.45 (0.13)	0.40 (0.12)	0.37 (0.13)	<.001
Social isolation scores, mean (*SD*)	0.84 (1.01)	0.87 (1.01)	0.84 (1.02)	0.82 (1.01)	.392

*Notes*: *SD* = standard deviation.

^a^Unless otherwise indicated, data are expressed as No. (%) of participants. Percentages have been rounded and may not total 100.

^b^A 1-way analysis of variance was used to compare the means of continuous variables; Pearson Chi-squared tests were performed to compare the distribution of categorical variables.

### The GS Loss Rate and the Cognitive Decline Rate

**Figure 1.**
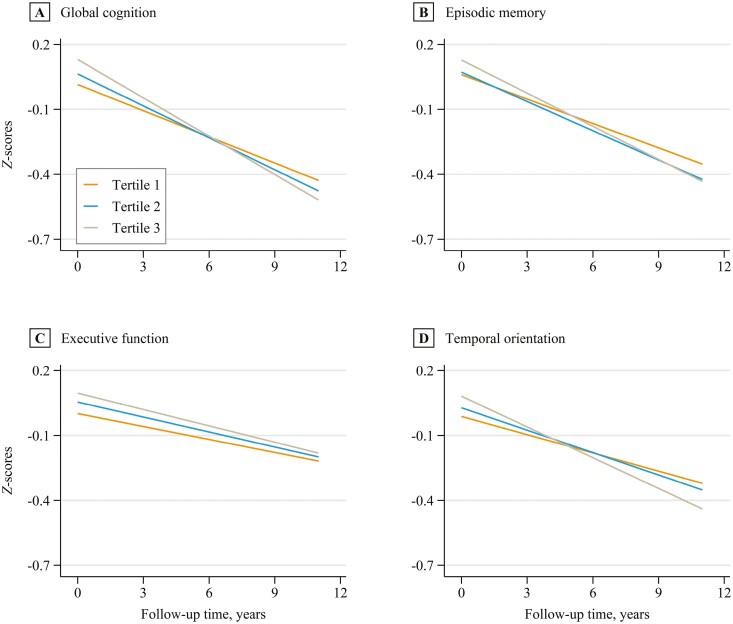
Predicted cognitive function *z* scores (standard deviation units) during Waves 4–9 by the tertiles of the absolute grip strength loss rate. Covariates were set to the following values: absolute grip strength = 31 kg, age = 68 y, women, White race, low-education level, retired, current nonsmokers, drinking for 1−4 d a week, moderate physical activity, overweight, social isolation scores = 1, not having depressive symptoms, hypertension, diabetes, and cardiovascular disease.

**Figure 2.**
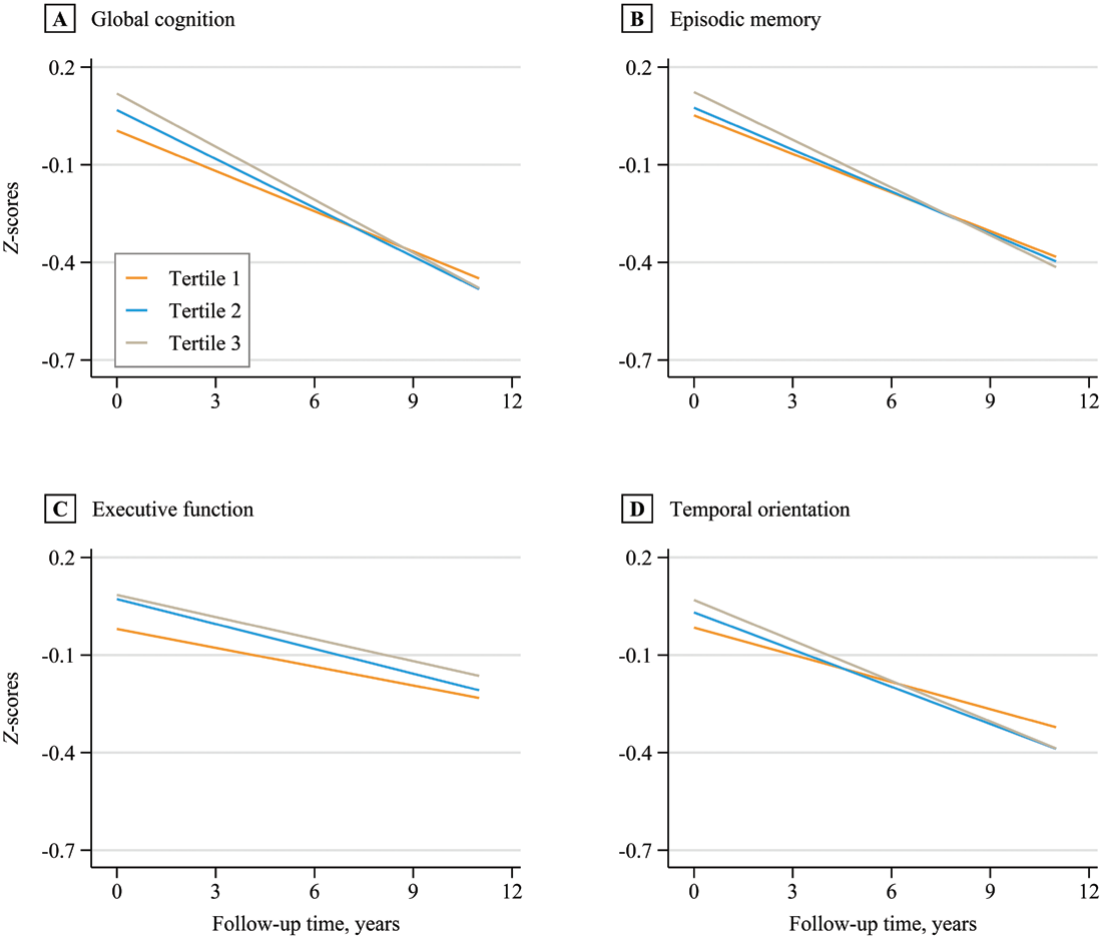
Predicted cognitive function *z* scores (standard deviation units) during Waves 4–9 by the tertiles of the relative grip strength loss rate. Covariates were set to the following values: relative grip strength = 0.40 kg/kg, age = 68 y, women, White race, low-education level, retired, current nonsmokers, drinking for 1−4 d a week, moderate physical activity, overweight, social isolation scores = 1, not having depressive symptoms, hypertension, diabetes, and cardiovascular disease.

As shown in Table 2, after adjusting for potential covariates, compared with Tertile 1 of the absolute GS loss rate, Tertile 2 was associated with a faster decline in global cognition (β = −0.009 [95% CI: −0.018 to −0.001] *SD*/year), but not in episodic memory (β = −0.004 [95% CI: −0.011 to 0.002] *SD*/year), executive function (β = −0.005 [95% CI: −0.011 to 0.002] *SD*/year) or temporal orientation (β = −0.010 [95% CI: −0.021to 0.002] *SD*/year). Compared with Tertile 1 of the absolute GS loss rate, Tertile 3 was correlated with a faster decline in global cognition (β = −0.018 [95% CI: −0.027 to −0.010]), episodic memory (β = −0.013 [95% CI: −0.019 to −0.006] *SD*/year), and temporal orientation (β = −0.021[95% CI: −0.033 to −0.009] *SD*/year), except for executive function (β = −0.004 [95% CI: −0.011 to 0.002] *SD*/year). Each 1 kg/year increase in the absolute GS loss rate was related to a faster decline in global cognition (β = −0.006 [95% CI: −0.009 to −0.004] *SD*/year), episodic memory (β = −0.004 [95% CI: −0.006 to −0.002] *SD*/year), and temporal orientation (β = −0.007 [95% CI: −0.011 to −0.004] *SD*/year), except for executive function (β = −0.002 [95% CI: −0.004 to 0.000] *SD*/year). The results of relative GS were similar to those of absolute GS. Figures 1 and 2 show predicted cognitive function *z* scores and 95% CIs during Waves 4–9 by absolute and relative GS loss rates, respectively.

**Table 2.** Association Between the Grip Strength Loss Rate During Waves 2–4 and Cognitive Decline Rates During Waves 4–9^a^

Variable	β coefficient (95% CI)			
	Absolute grip strength loss rate		Relative grip strength loss rate	
	Crude model	Adjusted model^b^	Crude model	Adjusted model^c^
Global cognition				
Tertile 1 × time	0.000 (Reference)	0.000 (Reference)	0.000 (Reference)	0.000 (Reference)
Tertile 2 × time	−0.009 (−0.018, −0.001)^*^	−0.009 (−0.018, −0.001)*	−0.009 (−0.017, −0.000)*	−0.009 (−0.017, −0.000)*
Tertile 3 × time	−0.018 (−0.027, −0.009)***	−0.018 (−0.027, −0.010)***	−0.013 (−0.021, −0.004)**	−0.013 (−0.022, −0.005)**
* p* Value for trend^d^	<.001	<.001	.007	.003
Per unit increment × time^e^	−0.006 (−0.009, −0.004)***	−0.006 (−0.009, −0.004)***	−0.359 (−0.542, −0.176)***	−0.366 (−0.548, −0.183)***
Episodic memory				
Tertile 1 × time	0.000 (Reference)	0.000 (Reference)	0.000 (Reference)	0.000 (Reference)
Tertile 2 × time	−0.004 (−0.011, 0.002)	−0.004 (−0.011, 0.002)	−0.003 (−0.010, 0.004)	−0.003 (−0.010, 0.003)
Tertile 3 × time	−0.012 (−0.019, −0.005)***	−0.013 (−0.019, −0.006)***	−0.009 (−0.016, −0.002)**	−0.009 (−0.016, −0.003)**
* p* Value for trend^d^	.003	<.001	.021	.007
Per unit increment × time^e^	−0.004 (−0.006, −0.002)***	−0.004 (−0.006, −0.002)***	−0.224 (−0.371, −0.077)**	−0.230 (−0.377, −0.084)**
Executive function				
Tertile 1 × time	0.000 (Reference)	0.000 (Reference)	0.000 (Reference)	0.000 (Reference)
Tertile 2 × time	−0.005 (−0.011, 0.002)	−0.005 (−0.011, 0.002)	−0.006 (−0.013, 0.001)	−0.006 (−0.013, 0.000)
Tertile 3 × time	−0.004 (−0.011, 0.002)	−0.004 (−0.011, 0.002)	−0.003 (−0.010, 0.003)	−0.003 (−0.010, 0.003)
* p* Value for trend^d^	.350	.202	.452	.322
Per unit increment × time^e^	−0.002 (−0.003, 0.000)	−0.002 (−0.004, 0.000)	−0.065 (−0.205, 0.075)	−0.069 (−0.209, 0.071)
Temporal orientation				
Tertile 1 × time	0.000 (Reference)	0.000 (Reference)	0.000 (Reference)	0.000 (Reference)
Tertile 2 × time	−0.010 (−0.021, 0.002)	−0.010 (−0.021, 0.002)	−0.010 (−0.022, 0.002)	−0.010 (−0.022, 0.001)
Tertile 3 × time	−0.021 (−0.033, −0.009)***	−0.021 (−0.033, −0.009)***	−0.013 (−0.025, −0.002)*	−0.014 (−0.025, −0.002)*
* p* Value for trend^d^	.002	<.001	.044	.022
Per unit increment × time^e^	−0.007 (−0.011, −0.004)***	−0.007 (−0.011, −0.004)***	−0.413 (−0.663, −0.163)**	−0.422 (−0.671, −0.172)***

*Notes*: ^a^The grip strength loss rate was divided into 3 groups according to the tertiles: <0.001, 0.001–0.979, and >0.979 kg/y for the absolute grip strength loss rate; <0.001, 0.001–0.013, and >0.013 kg/(kg × y) for the relative grip strength loss rate.

^b^Adjusted for age, sex, race/ethnicity, educational level, employment status, wealth, smoking status, drinking status, physical activity, body mass index, depressive symptoms, hypertension, diabetes, cardiovascular disease, social isolation score, and absolute grip strength in Wave 4.

^c^Adjusted for age, sex, race/ethnicity, educational level, employment status, wealth, smoking status, drinking status, physical activity, body mass index, depressive symptoms, hypertension, diabetes, cardiovascular disease, social isolation score, and relative grip strength in Wave 4.

^d^Test for linear trend was performed using the median grip strength loss rate for each tertile as a continuous variable.

^e^One kg/y for the absolute grip strength loss rate and 1 kg/(kg × y) for the relative grip strength loss rate.

^*^ *p* < .05; **p < .01; ***p < .001.

### The Moderating Role of Social Isolation

The moderating role of social isolation was assessed in the association of GS loss rates with decline rates in global cognition, episodic memory, and temporal orientation. As presented in Table 3, social isolation was a significant modifier in the relationship between absolute GS loss rate and decline rates in global cognition (β = −0.002 [95% CI: −0.005 to −0.001] *SD*/year for 3-way interaction) and episodic memory (β = −0.002 [95% CI: −0.004 to −0.001] *SD*/year for 3-way interaction), but not in temporal orientation (β = −0.002 [95% CI: −0.005 to 0.001] *SD*/year for 3-way interaction). However, we did not find a significant moderating role of social isolation in the correlation of the relative GS loss rate with decline rates in global cognition (β = −0.133 [95% CI: −0.304 to 0.038] *SD*/year for 3-way interaction), episodic memory (β = −0.102 [95% CI: −0.239 to 0.035] *SD*/year for 3-way interaction), or temporal orientation (β = −0.102 [95% CI: −0.335 to 0.131] *SD*/year for 3-way interaction).

**Table 3.** Modifying Role Of Social Isolation in Wave 4 in the Association Between the Grip Strength Loss Rate During Waves 2–4 and the Cognitive Decline Rate During Waves 4–9^a^

Variable	β coefficient (95% CI)			
	Absolute grip strength loss rate		Relative grip strength loss rate	
	Crude model	Adjusted model^b^	Crude model	Adjusted model^c^
Global cognition				
Per unit increment × social isolation × time^d^	−0.002 (−0.005, −0.001)*	−0.002 (−0.005, −0.001)*	−0.137 (−0.308, 0.034)	−0.133 (−0.304, 0.038)
Episodic memory				
Per unit increment × social isolation × time^d^	−0.002 (−0.004, −0.001)*	−0.002 (−0.004, −0.001)*	−0.103 (−0.240, 0.034)	−0.102 (−0.239, 0.035)
Temporal orientation				
Per unit increment × social isolation × time^d^	−0.002 (−0.005, 0.001)	−0.002 (−0.005, 0.001)	−0.103 (−0.336, 0.130)	−0.102 (−0.335, 0.131)

*Notes*: ^a^The grip strength loss rate was divided into 3 groups according to the tertiles: <0.001, 0.001–0.979, and >0.979 kg/y for the absolute grip strength loss rate; <0.001, 0.001–0.013, and >0.013 kg/(kg × y) for the relative grip strength loss rate.

^b^Adjusted for age, sex, race/ethnicity, educational level, employment status, wealth, smoking status, drinking status, physical activity, body mass index, depressive symptoms, hypertension, diabetes, cardiovascular disease, social isolation score, and absolute grip strength in Wave 4.

^c^Adjusted for age, sex, race/ethnicity, educational level, employment status, wealth, smoking status, drinking status, physical activity, body mass index, depressive symptoms, hypertension, diabetes, cardiovascular disease, social isolation score, and relative grip strength in Wave 4.

^d^One kg/y for the absolute grip strength loss rate and 1 kg/(kg × y) for the relative grip strength loss rate.

^*^ *p* < .05.

### Stratified Analysis by Age

The results in different age groups were almost consistent with those in the total sample. As shown in Supplementary Table 1, compared with Tertile 1 of the absolute GS loss rate, Tertile 3 was correlated with faster decline rates in global cognition (β = −0.019 [95% CI: −0.030 to −0.008] for 50–64 years; β = −0.013 [95% CI: −0.026 to −0.001] for 65–90 years), episodic memory (β = −0.014 [95% CI: −0.024 to −0.005] for 50–64 years; β = −0.009 [95% CI: −0.018 to −0.000] for 65–90 years), and temporal orientation (β = −0.018 [95% CI: −0.032 to −0.004] for 50–64 years; β = −0.023 [95% CI: −0.044 to −0.000] for 65–90 years), except for executive function. Each 1 kg/year increase in the absolute GS loss rate was related to a faster decline in global cognition (β = −0.006 [95% CI: −0.009 to −0.003] for 50–64 years; β = −0.006 [95% CI: −0.009 to −0.002] for 65–90 years), episodic memory (β = −0.003 [95% CI: −0.005 to −0.000] for 50–64 years; β = −0.004 [95% CI: −0.006 to −0.001] for 65–90 years), and temporal orientation (β = −0.006 [95% CI: −0.010 to −0.002] for 50–64 years; β = −0.007 [95% CI: −0.013 to −0.002] for 65–90 years), except for executive function. The results of the relative GS rate were similar to those of absolute GS. In 2 age groups, social isolation moderated the association between the absolute GS loss rate and decline rates in global cognition and episodic memory (Supplementary Table 2).

### Sensitivity Analyses

After excluding participants without children, the results were similar to those in the main analysis (Supplementary Tables 3 and 4). In the analysis adjusted for time-varying covariates, we also observed results similar to those of the main analysis (Supplementary Tables 5 and 6). After calculating relative GS through the ratio of absolute GS to body mass index, we found that the faster absolute GS loss rate was correlated with faster decline rates in global cognition, episodic memory, and temporal orientation, except for executive function (Supplementary Table 7). However, we did not find that social isolation moderated the associations of the relative GS loss rate with decline rates in global cognition, episodic memory, or temporal orientation (Supplementary Table 8).

## Discussion

Up to now, limited evidence is available on the association between the GS loss rate and the sequent cognitive decline rate in adults aged 50 years and older. Using the nationally representative data from the ELSA, this prospective cohort study found that faster absolute and relative GS losses might be associated with a faster subsequent cognitive decline rate, independent of baseline absolute and relative GS in older adults, respectively. Moreover, we also found that social isolation might moderate the association between the absolute GS loss rate and sequent cognitive decline rate. Our findings provide more nuanced insights for identifying high-risk individuals for rapid cognitive decline and formulating prevention strategies.

This study found that a faster GS loss rate was associated with a faster subsequent cognitive decline rate among adults aged 50 years and older in the United Kingdom, which is somewhat similar to the findings of previous studies (10–12). A longitudinal study conducted among Americans and Australians aged 65 years and older reported that a faster absolute GS loss rate was related to higher risks of dementia and cognitive impairment (10). Another longitudinal study carried out in South Korean adults aged 45 years and older showed that a faster absolute GS loss rate was correlated with lower cognitive function scores (11). Using data from 9 longitudinal studies, Zammit et al. found that a faster GS loss rate was associated with a faster cognitive decline rate among adults with an average age greater than 65 years old (12). In the previous studies (10–12), the GS loss rate and cognitive function were evaluated in the same period, so these studies cannot determine whether the GS loss rate can predict sequent cognitive function or the cognitive decline rate due to the possible bidirectional correlation (15,16). In addition, previous studies have focused on the absolute GS loss, ignoring the relative GS loss (10–12). By exploring the associations of absolute and relative GS loss rates during Waves 2–4 with the cognitive decline rate during Waves 4–9, this 10-year prospective cohort provides more robust evidence, filling the gap in previous studies. GS measurement is cheap, noninvasive, fast, and reliable, so the GS of older adults should be monitored for a long time to identify high-risk populations with rapid cognitive decline in the early stages.

Additionally, we also explored the associations between the GS loss rate and decline rates in 3 dimensions of cognitive function and found that absolute and relative GS loss rates were positively related to the decline rates in episodic memory and temporal orientation, but not to those in executive function. Previous studies have shown that a lower GS is associated with poorer episodic memory (38,39), but not with executive function (40,41), which to some extent supports our findings. Collectively, our findings indicate that a faster GS loss rate, in absolute and relative terms, might be associated with a faster decline rate in global cognition by affecting the performance of episodic memory and temporal orientation.

We observed that social isolation moderated the association of the absolute GS loss rate with the cognitive decline rate. Many studies have pointed out that reducing social isolation can help slow down cognitive decline among older adults (20,21,42). The biological mechanisms underlying cognitive decline caused by GS loss and social isolation both involve hippocampal volume atrophy (43–45) which may explain why low social isolation scores mitigate the association of the absolute GS loss rate with the cognitive decline rate. Thus, to prevent the rapid decline of cognitive function, older adults who have lost GS quickly should actively participate in some organizations (eg, religious groups, gyms/sports clubs, and committees), and their families should take the initiative to contact them regularly. It is noteworthy that the association between the relative GS loss rate and the cognitive decline rate was not moderated by social isolation. We speculate that the reason might be that some older adults lost relative GS due to weight gain. In such cases, social isolation cannot moderate the association of the relative GS loss rate with the cognitive decline rate. The exact reason needs to be clarified in future studies.

## Strengths and Limitations

This study has several important strengths. First, a prospective study design can identify the temporality between the GS loss rate and the cognitive decline rate, making up for the shortcomings of previous studies. Second, to account for variation in measurement time across individuals in the same wave, we evaluated the GS loss rate and the cognitive decline rate based on the survey month of each participant, making our results more reliable. Finally, in addition to global cognition, the outcomes included 3 dimensions of cognitive function (ie, episodic memory, executive function, and temporal orientation), which helps to clarify the specific dimensions of cognitive function affected by GS loss.

However, this study still has some limitations. First, cognitive function includes multiple dimensions, but this study only assessed episodic memory, executive function, and temporal orientation. A comprehensive cognitive function assessment is needed to explore the association of GS loss and social isolation with global cognitive decline. Second, previous studies have shown that cognitive decline might also cause GS loss (15,16). Although the assessment date of GS decline is earlier than that of cognitive decline in this study, the possibility of reverse causation cannot be fully eliminated. Third, because our sample was restricted to the general population of older people in the United Kingdom, the results might not be generalized to other countries. Our findings need to be validated in different populations. Finally, due to the nature of observational studies, the impact of unmeasured confounding factors (eg, APOE genotype) on our results cannot be eliminated, hindering the determination of the causal association.

## Conclusion

In conclusion, both faster absolute and relative GS loss rates were associated with a faster sequent cognitive decline rate, independent of baseline absolute and relative GS in older adults, respectively. Low social isolation scores attenuated the association of the absolute GS loss rate with the cognitive decline rate. The routine monitoring of GS should be recommended among older adults, and those who have rapidly lost absolute or relative GS might be the high-risk population with rapid cognitive decline. Older adults should actively participate in physical exercise to reduce GS loss, and those who have lost GS quickly should actively participate in more social activities.

## Supplementary Material

igae055_suppl_Supplementary_Materials

## Data Availability

The data sets will be available from the corresponding author upon reasonable request. This study was not preregistered.
