# Supplementary material for: The Grip Strength Loss Rate and the Subsequent Cognitive Decline Rate in Older Adults: The Moderating Role of Social Isolation
Source: Innov Aging. 2024 Jun 4;8(8):igae055. doi: 10.1093/geroni/igae055 (PMC11322675; doi:10.1093/geroni/igae055)
Supplement: igae055_suppl_Supplementary_Materials [file igae055_suppl_supplementary_materials.docx]

***Innovation in Aging* Supplementary Material: Li et al. The grip strength loss rate and the subsequent cognitive decline rate in older adults: the moderating role of social isolation.**

**Table S1.** Stratified analysis by age: association between the rate of grip strength loss during waves 2–4 and the rate of cognitive decline during waves 4–9.

**Table S2.** Stratified analysis by age: modifying role of social isolation in wave 4 in the association between the rate grip strength loss during waves 2–4 and the rate of cognitive decline during waves 4–9.

**Table S3.** Sensitivity analyses of the association between the rate of grip strength loss during waves 2–4 and the rate of cognitive decline during waves 4–9: excluding participants without children.

**Table S4.** Sensitivity analyses of the modifying role of social isolation in wave 4 in the association between the rate of grip strength loss during waves 2–4 and the rate of cognitive decline during waves 4–9: excluding participants without children.

**Table S5.** Sensitivity analyses of the association between the rate of grip strength loss during waves 2–4 and the rate of cognitive decline during waves 4–9: time-varying covariates.

**Table S6.** Sensitivity analyses of the modifying role of social isolation in wave 4 in the association between the rate of grip strength loss during waves 2–4 and the rate of cognitive decline during waves 4–9: time-varying covariates.

**Table S7.** Sensitivity analyses of the association between the rate of relative grip strength loss during waves 2–4 and the rate of cognitive decline during waves 4–9: relative grip strength = grip strength / body mass index.

**Table S8.** Sensitivity analyses of the modifying role of social isolation in wave 4 in the association between the rate of grip strength loss during waves 2–4 and the rate of cognitive decline during waves 4–9: relative grip strength = grip strength / body mass index.

**Figure S1.** The timeline and cumulative attrition rates of this study.

**Figure S2.** The inclusion and exclusion process of participants in this study.

| Table S1. Stratified analysis by age: association between the rate of grip strength loss during waves 2–4 and the rate of cognitive decline during waves 4–9. | | | | | |
| --- | --- | --- | --- | --- | --- |
|  | ***β* coefficient (95% CI)** | | | | |
|  | **Absolute grip strength** **loss^a^** | |  | **Relative grip strength loss^b^** | |
|  | **50–64 years** | **65–90 years** |  | **50–64 years** | **65–90 years** |
| **Global cognition** |  |  |  |  |  |
| Tertile 1 × time | 0.000 (Reference) | 0.000 (Reference) |  | 0.000 (Reference) | 0.000 (Reference) |
| Tertile 2 × time | -0.008 (-0.019, 0.003) | -0.005 (-0.017, 0.007) |  | -0.011 (-0.022, -0.001)^*^ | -0.004 (-0.017, 0.008) |
| Tertile 3 × time | -0.019 (-0.030, -0.008)^‡^ | -0.013 (-0.026, -0.001)^*^ |  | -0.018 (-0.029, -0.007)^†^ | -0.008 (-0.017, -0.000)^*^ |
| *P*-value for trend^c^ | .005 | .03 |  | .002 | .03 |
| Per unit increment × time^d^ | -0.006 (-0.009, -0.003)^‡^ | -0.006 (-0.009, -0.002)^†^ |  | -0.396 (-0.632, -0.160)^†^ | -0.290 (-0.557, -0.023)^*^ |
| **Episodic memory** |  |  |  |  |  |
| Tertile 1 × time | 0.000 (Reference) | 0.000 (Reference) |  | 0.000 (Reference) | 0.000 (Reference) |
| Tertile 2 × time | -0.006 (-0.016, 0.003) | -0.005 (-0.015, 0.004) |  | -0.007 (-0.017, 0.002) | -0.001 (-0.010, 0.009) |
| Tertile 3 × time | -0.014 (-0.024, -0.005)^†^ | -0.009 (-0.018, -0.000)^*^ |  | -0.014 (-0.023, -0.004)^†^ | -0.006 (-0.013, -0.000)^*^ |
| *P*-value for trend^c^ | .008 | .045 |  | .01 | .04 |
| Per unit increment × time^d^ | -0.003 (-0.005, -0.000)^†^ | -0.004 (-0.006, -0.001)^†^ |  | -0.229 (-0.436, -0.021)^*^ | -0.194 (-0.398, -0.001)^*^ |
| **Executive function** |  |  |  |  |  |
| Tertile 1 × time | 0.000 (Reference) | 0.000 (Reference) |  | 0.000 (Reference) | 0.000 (Reference) |
| Tertile 2 × time | -0.005 (-0.015, 0.004) | 0.002 (-0.007, 0.011) |  | -0.009 (-0.019, 0.000) | -0.002 (-0.011, 0.007) |
| Tertile 3 × time | -0.007 (-0.017, 0.002) | -0.000 (-0.009, 0.009) |  | -0.010 (-0.020, 0.000) | 0.006 (-0.003, 0.015) |
| *P*-value for trend^c^ | .26 | 0.95 |  | .05 | .33 |
| Per unit increment × time^d^ | -0.003 (-0.005, 0.000) | 0.000 (-0.003, 0.003) |  | -0.174 (-0.378, 0.029) | 0.051 (-0.140, 0.242) |
| **Temporal orientation** |  |  |  |  |  |
| Tertile 1 × time | 0.000 (Reference) | 0.000 (Reference) |  | 0.000 (Reference) | 0.000 (Reference) |
| Tertile 2 × time | -0.007 (-0.021, 0.007) | -0.003 (-0.021, 0.014) |  | -0.009 (-0.023, 0.005) | -0.008 (-0.026, 0.009) |
| Tertile 3 × time | -0.018 (-0.032, -0.004)^†^ | -0.023 (-0.044, -0.000)^*^ |  | -0.014 (-0.028, -0.000)^*^ | -0.013 (-0.026, -0.001)^*^ |
| *P*-value for trend^c^ | .03 | .047 |  | .03 | .049 |
| Per unit increment × time^d^ | -0.006 (-0.010, -0.002)^†^ | -0.007 (-0.013, -0.002)^†^ |  | -0.412 (-0.713, -0.112)^†^ | -0.396 (-0.775, -0.017)^*^ |
| * *P*-value <.05; † *P*-value <.01; ‡ *P*-value <.001.  ^a^ Adjusted for age, sex, race/ethnicity, education level, employment status, wealth, smoking status, drinking status, physical activity, body mass index, depressive symptoms, hypertension, diabetes, cardiovascular disease, social isolation score, and absolute grip strength in wave 4.  ^b^ Adjusted for age, sex, race/ethnicity, education level, employment status, wealth, smoking status, drinking status, physical activity, body mass index, depressive symptoms, hypertension, diabetes, cardiovascular disease, social isolation score, and relative grip strength in wave 4.  ^c^ Test for linear trend was performed using the median grip strength loss for each tertile as a continuous variable.  ^d^ One kg/year for the rate of absolute grip strength lose and 1 kg/(kg × year) for the rate of relative grip strength lose. | | | | | |

| Table S2. Stratified analysis by age: modifying role of social isolation in wave 4 in the association between the rate grip strength loss during waves 2–4 and the rate of cognitive decline during waves 4–9. | | | | | |
| --- | --- | --- | --- | --- | --- |
|  | ***β* coefficient (95% CI)** | | | | |
|  | **Absolute grip strength loss^a^** | |  | **Relative grip strength loss^b^** | |
|  | **50–64 years old** | **65–90 years old** |  | **50–64 years old** | **65–90 years old** |
| **Global cognition** |  |  |  |  |  |
| Per unit increment × social isolation × time^c^ | -0.002 (-0.004, -0.001)^*^ | -0.003 (-0.006, -0.000)^*^ |  | -0.133 (-0.304, 0.038) | -0.180 (-0.412, 0.052) |
| **Episodic memory** |  |  |  |  |  |
| Per unit increment × social isolation × time^c^ | -0.001 (-0.002, -0.001)^*^ | -0.002 (-0.005, -0.000)^*^ |  | -0.012 (-0.227, 0.203) | -0.161 (-0.338, 0.016) |
| **Temporal orientation** |  |  |  |  |  |
| Per unit increment × social isolation × time^c^ | -0.000 (-0.005, 0.004) | -0.003 (-0.008, 0.001) |  | -0.021 (-0.333, 0.290) | -0.169 (-0.499, 0.160) |
| ^*^ *P*-value <.05.  ^a^ Adjusted for age, sex, race/ethnicity, education level, employment status, wealth, smoking status, drinking status, physical activity, body mass index, depressive symptoms, hypertension, diabetes, cardiovascular disease, social isolation score, and absolute grip strength in wave 4.  ^b^ Adjusted for age, sex, race/ethnicity, education level, employment status, wealth, smoking status, drinking status, physical activity, body mass index, depressive symptoms, hypertension, diabetes, cardiovascular disease, social isolation score, and relative grip strength in wave 4.  ^c^ One kg/year for the rate of absolute grip strength lose and 1 kg/(kg × year) for the rate of relative grip strength lose. | | | | | |

| Table S3. Sensitivity analyses of the association between the rate of grip strength loss during waves 2–4 and the rate of cognitive decline during waves 4–9: excluding participants without children. | | |
| --- | --- | --- |
|  | ***β* coefficient (95% CI)** |  |
|  | **Absolute grip strength loss^a^** | **Relative grip strength loss^b^** |
| Global cognition |  |  |
| Tertile 1 × time | 0.000 (Reference) | 0.000 (Reference) |
| Tertile 2 × time | -0.009 (-0.018, 0.000) | -0.009 (-0.017, 0.000) |
| Tertile 3 × time | -0.019 (-0.028, -0.010)^‡^ | -0.014 (-0.023, -0.005)^†^ |
| *P*-value for trend^c^ | <.001 | .004 |
| Per unit increment × time^d^ | -0.006 (-0.009, -0.004)^‡^ | -0.353 (-0.546, -0.159)^‡^ |
| Episodic memory |  |  |
| Tertile 1 × time | 0.000 (Reference) | 0.000 (Reference) |
| Tertile 2 × time | -0.008 (-0.015, -0.001)^*^ | -0.005 (-0.012, 0.002) |
| Tertile 3 × time | -0.014 (-0.021, -0.007)^‡^ | -0.010 (-0.017, -0.003)^†^ |
| *P*-value for trend^c^ | <.001 | .007 |
| Per unit increment × time^d^ | -0.004 (-0.006, -0.002)^‡^ | -0.219 (-0.373, -0.064)^†^ |
| Executive function |  |  |
| Tertile 1 × time | 0.000 (Reference) | 0.000 (Reference) |
| Tertile 2 × time | -0.002 (-0.009, 0.005) | -0.006 (-0.013, 0.001) |
| Tertile 3 × time | -0.004 (-0.011, 0.003) | -0.002 (-0.008, 0.005) |
| *P*-value for trend^c^ | .33 | .66 |
| Per unit increment × time^d^ | -0.001 (-0.003, 0.001) | -0.033 (-0.181, 0.115) |
| Temporal orientation |  |  |
| Tertile 1 × time | 0.000 (Reference) | 0.000 (Reference) |
| Tertile 2 × time | -0.006 (-0.018, 0.006) | -0.009 (-0.021, 0.003) |
| Tertile 3 × time | -0.020 (-0.032, -0.008)^†^ | -0.016 (-0.028, -0.004)^†^ |
| *P*-value for trend^c^ | <.001 | .01 |
| Per unit increment × time^d^ | -0.007 (-0.011, -0.004)^‡^ | -0.444 (-0.707, -0.181)^‡^ |
| * *P*-value <.05; † *P*-value <.01; ‡ *P*-value <.001.  ^a^ Adjusted for age, sex, race/ethnicity, education level, employment status, wealth, smoking status, drinking status, physical activity, body mass index, depressive symptoms, hypertension, diabetes, cardiovascular disease, social isolation score, and absolute grip strength in wave 4.  ^b^ Adjusted for age, sex, race/ethnicity, education level, employment status, wealth, smoking status, drinking status, physical activity, body mass index, depressive symptoms, hypertension, diabetes, cardiovascular disease, social isolation score, and relative grip strength in wave 4.  ^c^ Test for linear trend was performed using the median grip strength loss for each tertile as a continuous variable.  ^d^ One kg/year for the rate of absolute grip strength lose and 1 kg/(kg × year) for the rate of relative grip strength lose. | | |

| Table S4. Sensitivity analyses of the modifying role of social isolation in wave 4 in the association between the rate of grip strength loss during waves 2–4 and the rate of cognitive decline during waves 4–9: excluding participants without children. | | |
| --- | --- | --- |
|  | ***β* coefficient (95% CI)** |  |
|  | **Absolute grip strength loss^a^** | **Relative grip strength loss^b^** |
| Global cognition |  |  |
| Per unit increment × social isolation × time^c^ | -0.002 (-0.005, 0.000)^*^ | -0.131 (-0.307, 0.045) |
| Episodic memory |  |  |
| Per unit increment × social isolation × time^c^ | -0.002 (-0.003, 0.000)^*^ | -0.088 (-0.229, 0.053) |
| Temporal orientation |  |  |
| Per unit increment × social isolation × time^c^ | -0.002 (-0.005, 0.001) | -0.108 (-0.347, 0.131) |
| * *P*-value <.05.  ^a^ Adjusted for age, sex, race/ethnicity, education level, employment status, wealth, smoking status, drinking status, physical activity, body mass index, depressive symptoms, hypertension, diabetes, cardiovascular disease, social isolation score, and absolute grip strength in wave 4.  ^b^ Adjusted for age, sex, race/ethnicity, education level, employment status, wealth, smoking status, drinking status, physical activity, body mass index, depressive symptoms, hypertension, diabetes, cardiovascular disease, social isolation score, and relative grip strength in wave 4.  ^c^ One kg/year for the rate of absolute grip strength lose and 1 kg/(kg × year) for the rate of relative grip strength lose. | | |

| Table S5. Sensitivity analyses of the association between the rate of grip strength loss during waves 2–4 and the rate of cognitive decline during waves 4–9: time-varying covariates. | | |
| --- | --- | --- |
|  | ***β* coefficient (95% CI)** |  |
|  | **Absolute grip strength loss^a^** | **Relative grip strength loss^b^** |
| Global cognition |  |  |
| Tertile 1 × time | 0.000 (Reference) | 0.000 (Reference) |
| Tertile 2 × time | -0.009 (-0.017, -0.000)^*^ | -0.008 (-0.016, 0.000) |
| Tertile 3 × time | -0.016 (-0.024, -0.008)^‡^ | -0.012 (-0.020, -0.004)^†^ |
| *P*-value for trend^c^ | <.001 | .004 |
| Per unit increment × time^d^ | -0.005 (-0.008, -0.003)^‡^ | -0.356 (-0.537, -0.175)^‡^ |
| Episodic memory |  |  |
| Tertile 1 × time | 0.000 (Reference) | 0.000 (Reference) |
| Tertile 2 × time | -0.007 (-0.014, -0.000)^*^ | -0.004 (-0.011, 0.003) |
| Tertile 3 × time | -0.012 (-0.019, -0.005)^‡^ | -0.009 (-0.016, -0.002)^*^ |
| *P*-value for trend^c^ | .001 | .02 |
| Per unit increment × time^d^ | -0.003 (-0.005, -0.001)^†^ | -0.217 (-0.368, -0.067)^†^ |
| Executive function |  |  |
| Tertile 1 × time | 0.000 (Reference) | 0.000 (Reference) |
| Tertile 2 × time | -0.003 (-0.009, 0.004) | -0.006 (-0.012, 0.001) |
| Tertile 3 × time | -0.004 (-0.011, 0.002) | -0.003 (-0.009, 0.004) |
| *P*-value for trend^c^ | .27 | .29 |
| Per unit increment × time^d^ | -0.001 (-0.003, 0.001) | -0.076 (-0.218, 0.067) |
| Temporal orientation |  |  |
| Tertile 1 × time | 0.000 (Reference) | 0.000 (Reference) |
| Tertile 2 × time | -0.007 (-0.018, 0.004) | -0.009 (-0.020, 0.002) |
| Tertile 3 × time | -0.016 (-0.027, -0.005)^†^ | -0.012 (-0.023, -0.001)^*^ |
| *P*-value for trend^c^ | .001 | .03 |
| Per unit increment × time^d^ | -0.006 (-0.009, -0.003)^‡^ | -0.405 (-0.646, -0.163)^†^ |
| * *P*-value <.05; † *P*-value <.01; ‡ *P*-value <.001.  ^a^ Adjusted for age, sex, race/ethnicity, education level, employment status, wealth, smoking status, drinking status, physical activity, body mass index, depressive symptoms, hypertension, diabetes, cardiovascular disease, social isolation score, and absolute grip strength in wave 4.  ^b^ Adjusted for age, sex, race/ethnicity, education level, employment status, wealth, smoking status, drinking status, physical activity, body mass index, depressive symptoms, hypertension, diabetes, cardiovascular disease, social isolation score, and relative grip strength in wave 4.  ^c^ Test for linear trend was performed using the median grip strength loss for each tertile as a continuous variable.  ^d^ One kg/year for the rate of absolute grip strength lose and 1 kg/(kg × year) for the rate of relative grip strength lose. | | |

| *Table S6.* Sensitivity analyses of the modifying role of social isolation in wave 4 in the association between the rate of grip strength loss during waves 2–4 and the rate of cognitive decline during waves 4–9: time-varying covariates. | | |
| --- | --- | --- |
|  | ***β* coefficient (95% CI)** |  |
|  | **Absolute grip strength loss^a^** | **Relative grip strength loss^b^** |
| Global cognition |  |  |
| Per unit increment × social isolation × time^c^ | -0.002 (-0.005, -0.000)^*^ | -0.002 (-0.005, 0.001) |
| Episodic memory |  |  |
| Per unit increment × social isolation × time^c^ | -0.001 (-0.002, -0.000)^*^ | 0.050 (-0.142, 0.243) |
| Temporal orientation |  |  |
| Per unit increment × social isolation × time^c^ | -0.001 (-0.005, 0.003) | -0.066 (-0.370, 0.237) |
| * *P*-value <.05.  ^a^ Adjusted for age, sex, race/ethnicity, education level, employment status, wealth, smoking status, drinking status, physical activity, body mass index, depressive symptoms, hypertension, diabetes, cardiovascular disease, social isolation score, and absolute grip strength in wave 4.  ^b^ Adjusted for age, sex, race/ethnicity, education level, employment status, wealth, smoking status, drinking status, physical activity, body mass index, depressive symptoms, hypertension, diabetes, cardiovascular disease, social isolation score, and relative grip strength in wave 4.  ^c^ One kg/year for the rate of absolute grip strength lose and 1 kg/(kg × year) for the rate of relative grip strength lose. | | |

| **Table S7.** Sensitivity analyses of the association between the rate of relative grip strength loss during waves 2–4 and the rate of cognitive decline during waves 4–9: relative grip strength = grip strength / body mass index. | |
| --- | --- |
|  | ***β* coefficient (95% CI)a** |
| Global cognition |  |
| Tertile 1 × time | 0.000 (Reference) |
| Tertile 2 × time | -0.008 (-0.016, 0.001) |
| Tertile 3 × time | -0.015 (-0.023, -0.006)^‡^ |
| *P*-value for trend^b^ | 0.001 |
| Per unit increment × time^c^ | -0.132 (-0.197, -0.068)^‡^ |
| Episodic memory |  |
| Tertile 1 × time | 0.000 (Reference) |
| Tertile 2 × time | -0.006 (-0.013, 0.001) |
| Tertile 3 × time | -0.010 (-0.017, -0.003)^*^ |
| *P*-value for trend^b^ | 0.004 |
| Per unit increment × time^c^ | -0.082 (-0.135, -0.030)^*^ |
| Executive function |  |
| Tertile 1 × time | 0.000 (Reference) |
| Tertile 2 × time | -0.004 (-0.010, 0.003) |
| Tertile 3 × time | -0.003 (-0.009, 0.004) |
| *P*-value for trend^b^ | 0.392 |
| Per unit increment × time^c^ | -0.029 (-0.079, 0.021) |
| Temporal orientation |  |
| Tertile 1 × time | 0.000 (Reference) |
| Tertile 2 × time | -0.010 (-0.021, 0.002) |
| Tertile 3 × time | -0.016 (-0.027, -0.004)^*^ |
| *P*-value for trend^b^ | 0.008 |
| Per unit increment × time^c^ | -0.149 (-0.237, -0.061)^‡^ |
| * *P*-value <.05.  † *P*-value <.01.  ‡ *P*-value <.001.  ^a^ Adjusted for age, sex, race/ethnicity, education level, employment status, wealth, smoking status, drinking status, physical activity, body mass index, depressive symptoms, hypertension, diabetes, cardiovascular disease, social isolation score, and relative grip strength in wave 4.  ^b^ Test for linear trend was performed using the median relative grip strength loss for each tertile as a continuous variable.  ^c^ One kg/(kg/m^2^ × year) for the rate of relative grip strength lose. | |

| **Table S8.** Sensitivity analyses of the modifying role of social isolation in wave 4 in the association between the rate of grip strength loss during waves 2–4 and the rate of cognitive decline during waves 4–9: relative grip strength = grip strength / body mass index. | |
| --- | --- |
|  | ***β* coefficient (95% CI)^a^** |
| Global cognition |  |
| Per unit increment × social isolation × time^b^ | -0.057 (-0.118, 0.004) |
| Episodic memory |  |
| Per unit increment × social isolation × time^b^ | -0.038 (-0.087, 0.012) |
| Temporal orientation |  |
| Per unit increment × social isolation × time^b^ | -0.046 (-0.129, 0.037) |
| ^a^ Adjusted for age, sex, race/ethnicity, education level, employment status, wealth, smoking status, drinking status, physical activity, depressive symptoms, hypertension, diabetes, cardiovascular disease, social isolation score, and relative grip strength in wave 4.  ^b^ One kg/year for the rate of absolute grip strength lose and 1 kg/(kg × year) for the rate of relative grip strength lose. | |


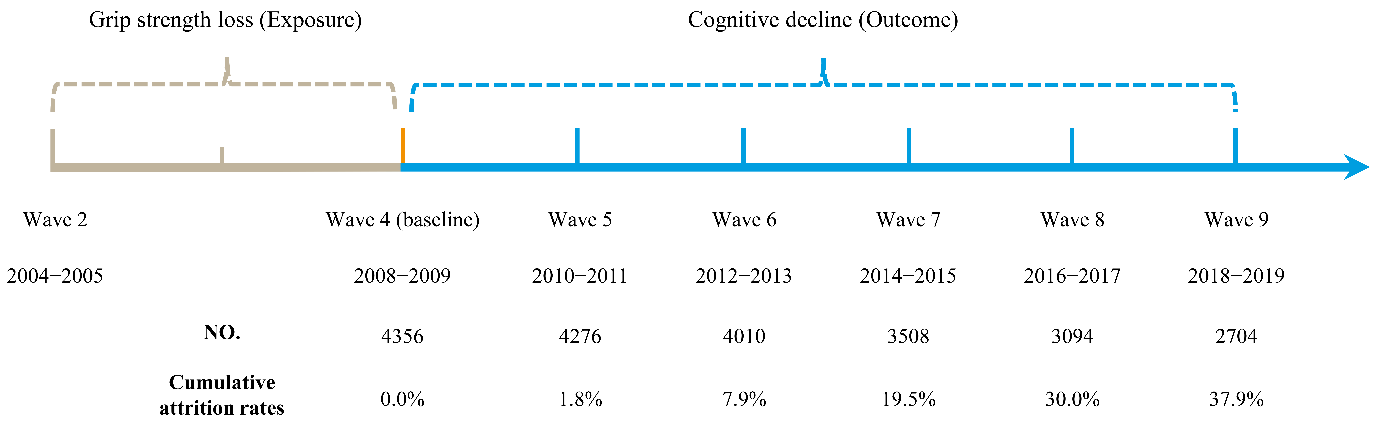


Figure S1. The timeline and cumulative attrition rates of this study.

Grip strength loss was measured in waves 2 and 4, and cognitive decline was assessed during waves 4–9.


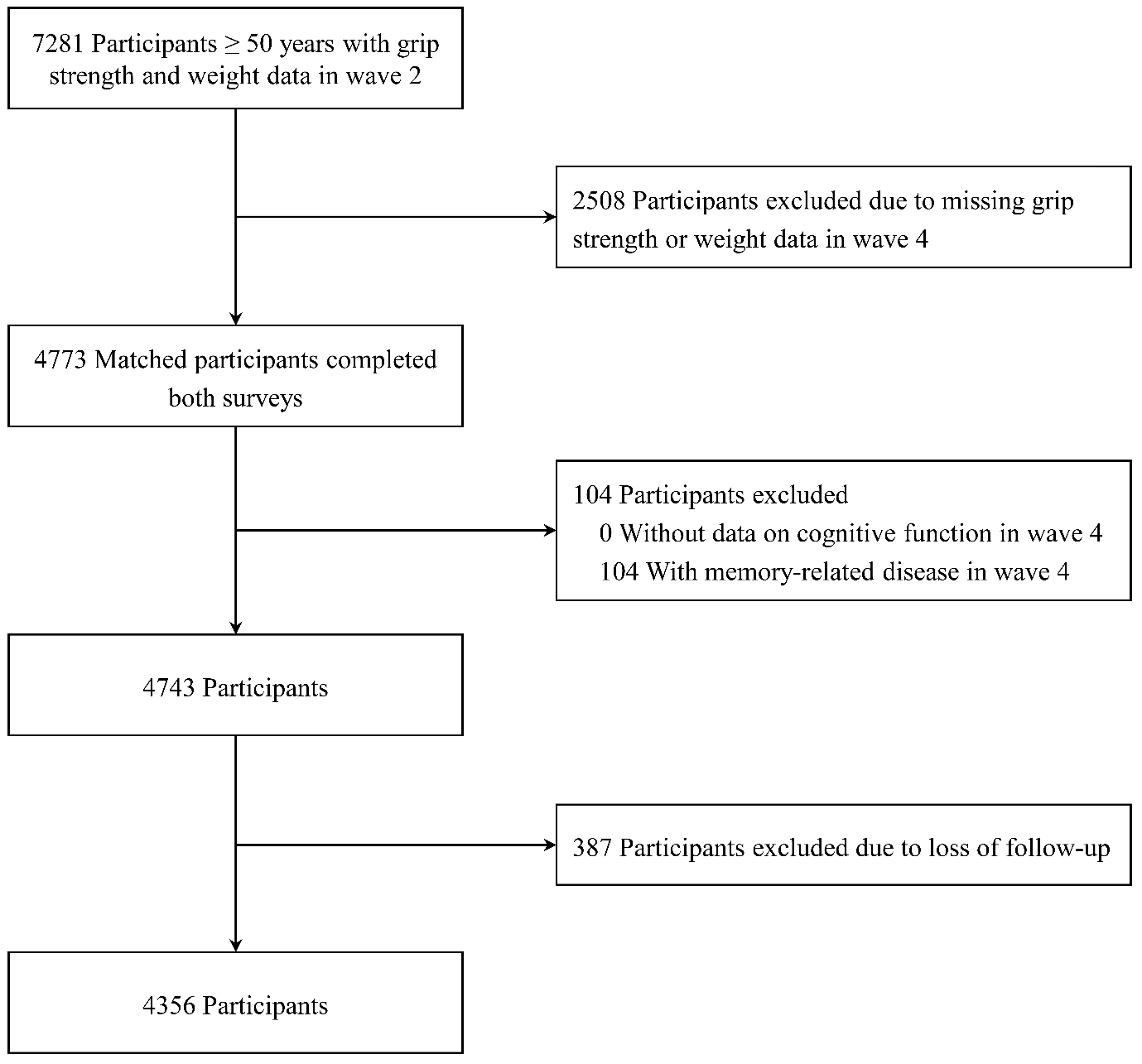


Figure S2. The inclusion and exclusion process of participants in this study.
